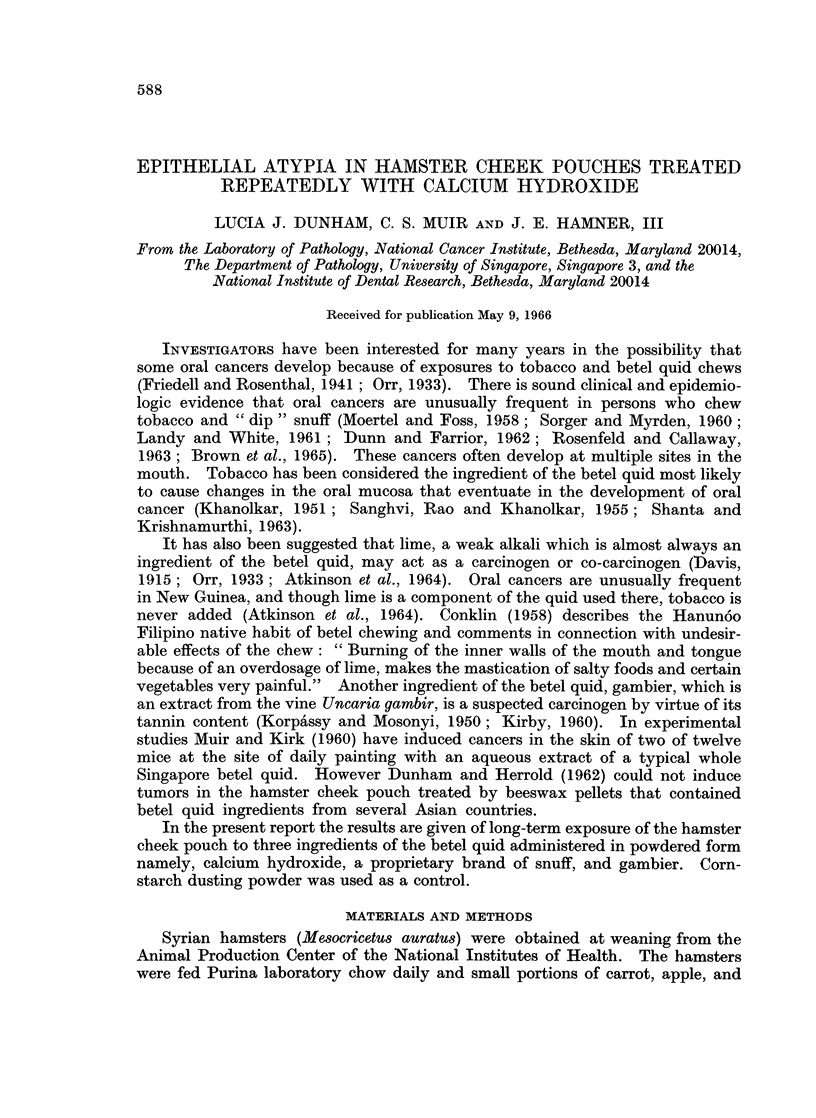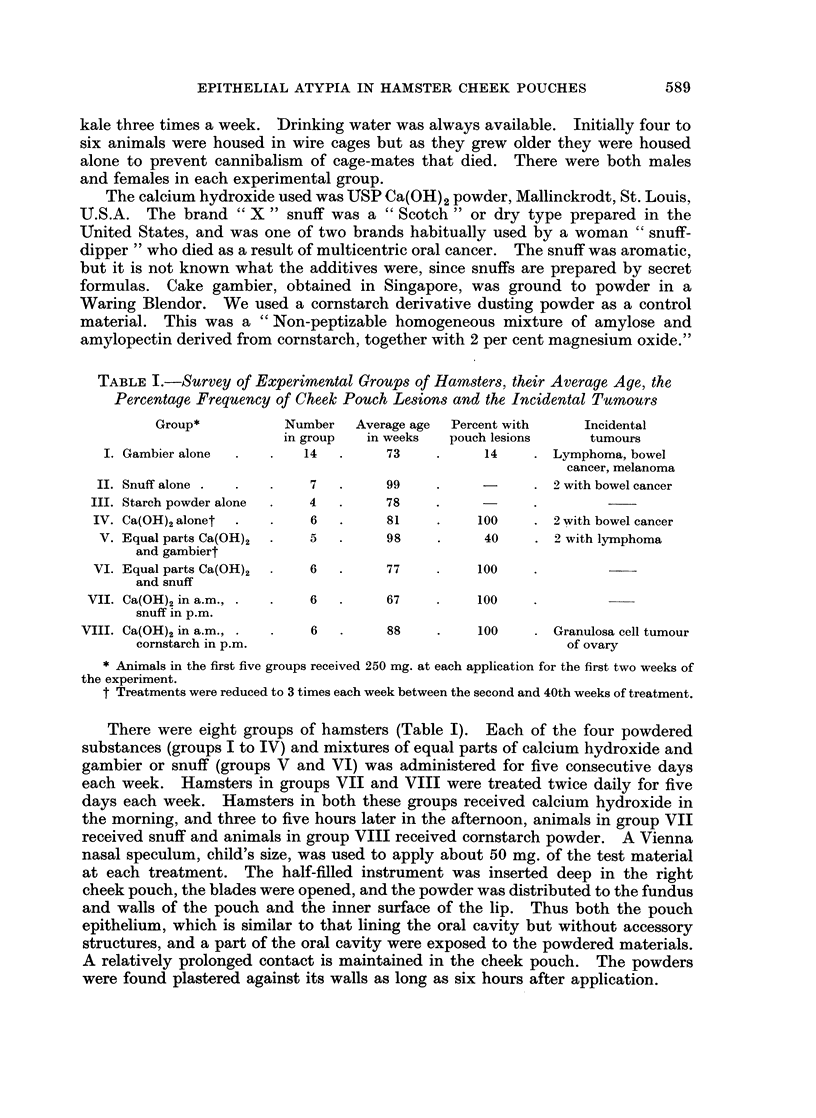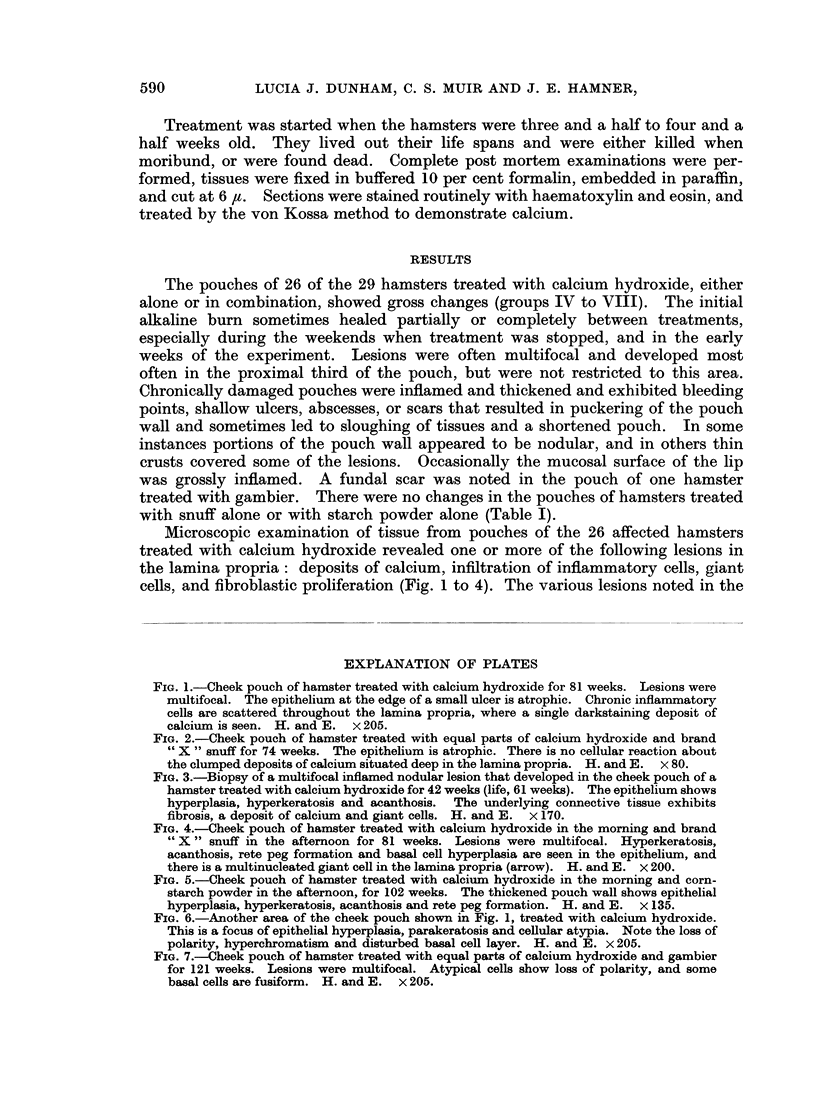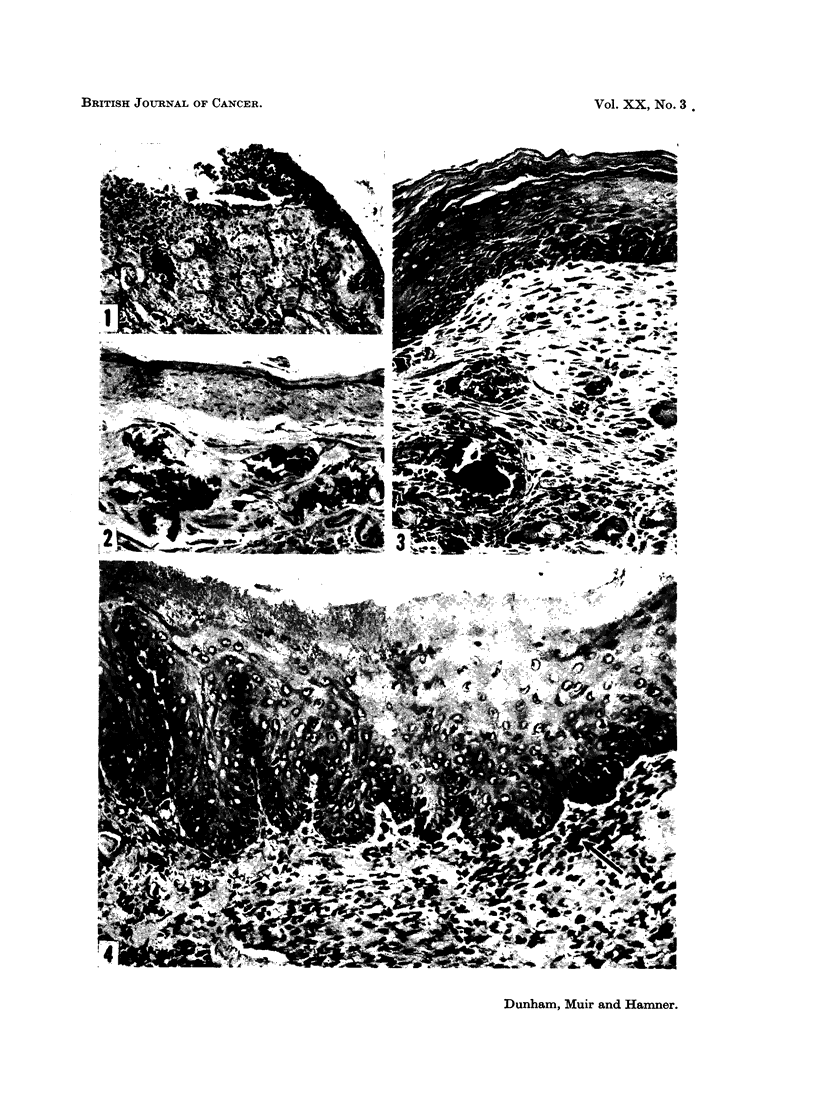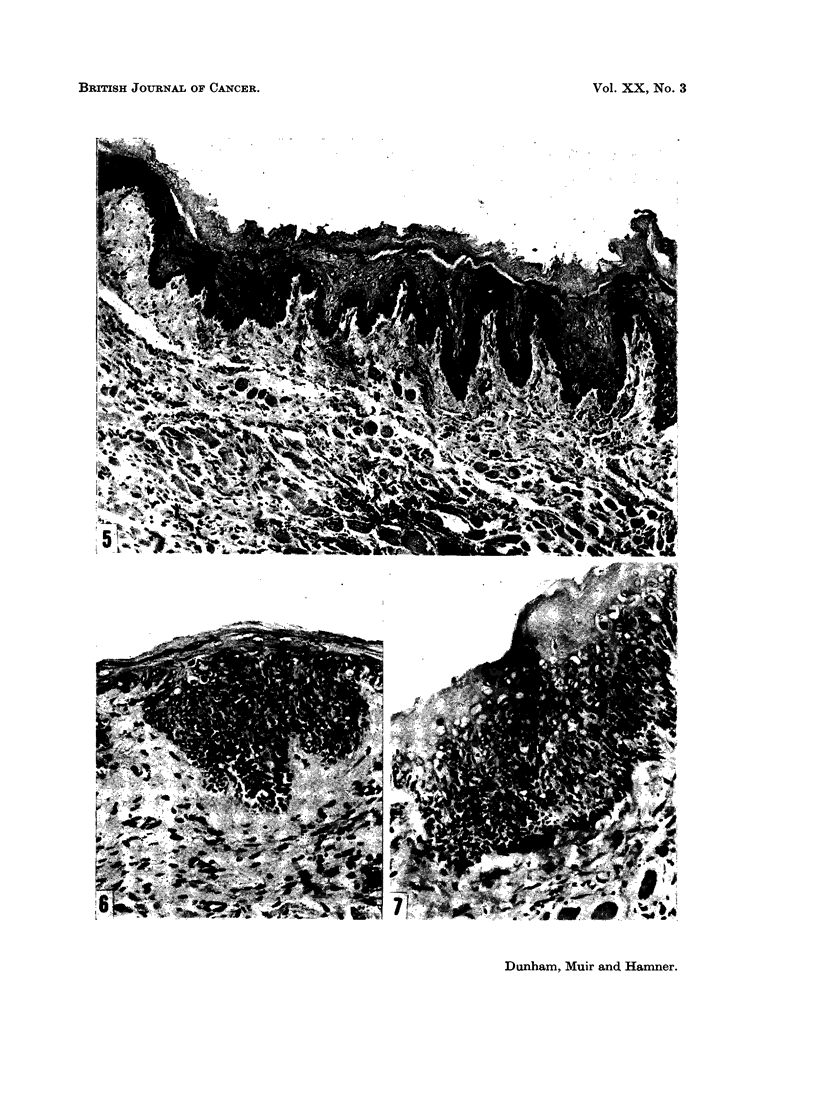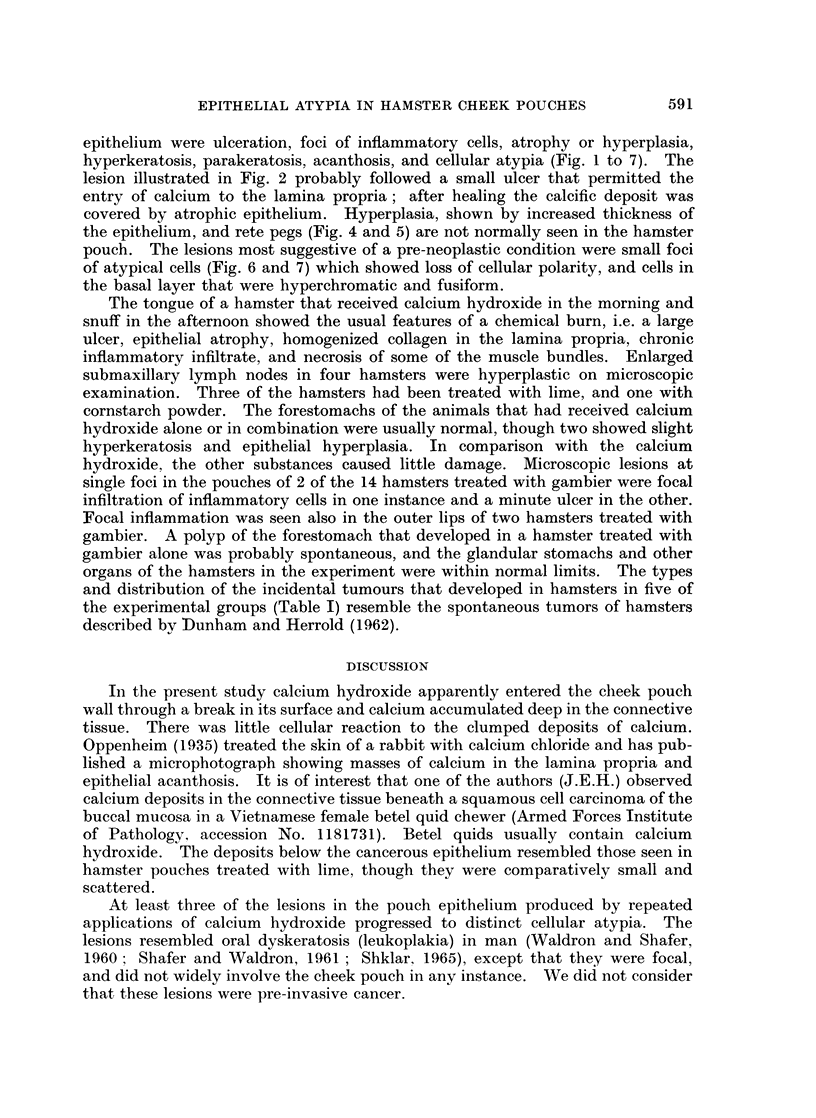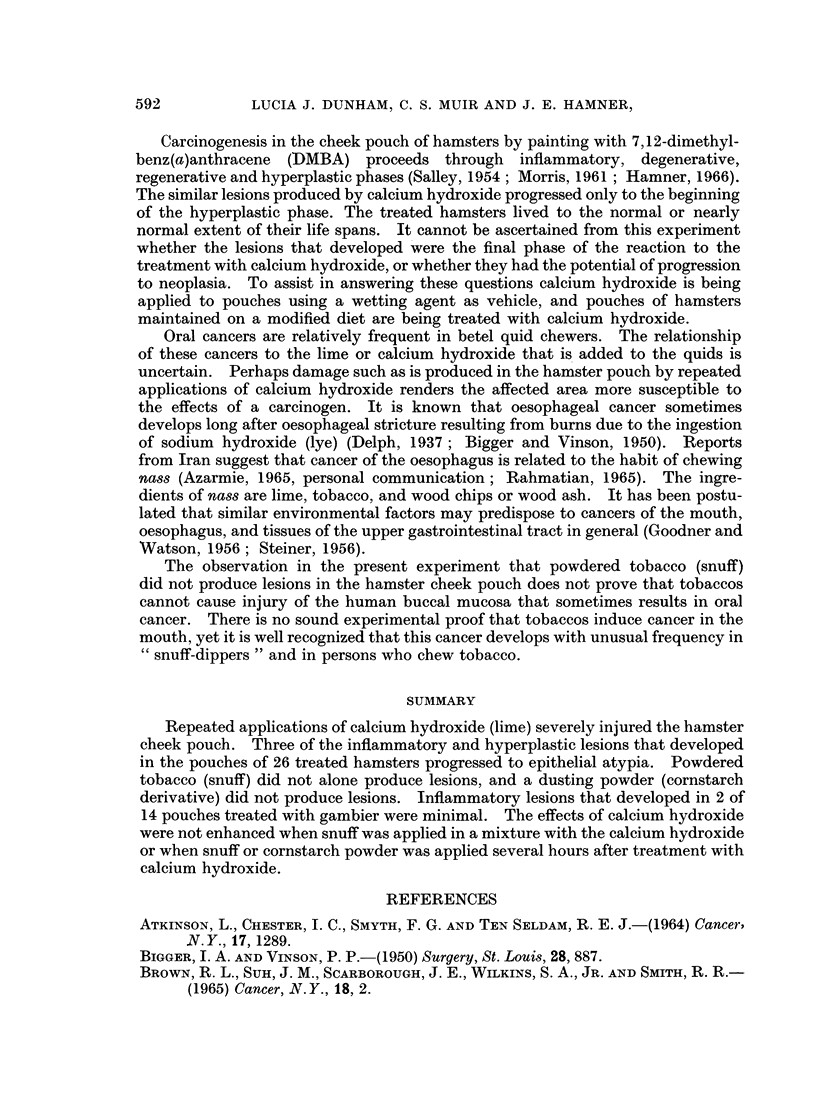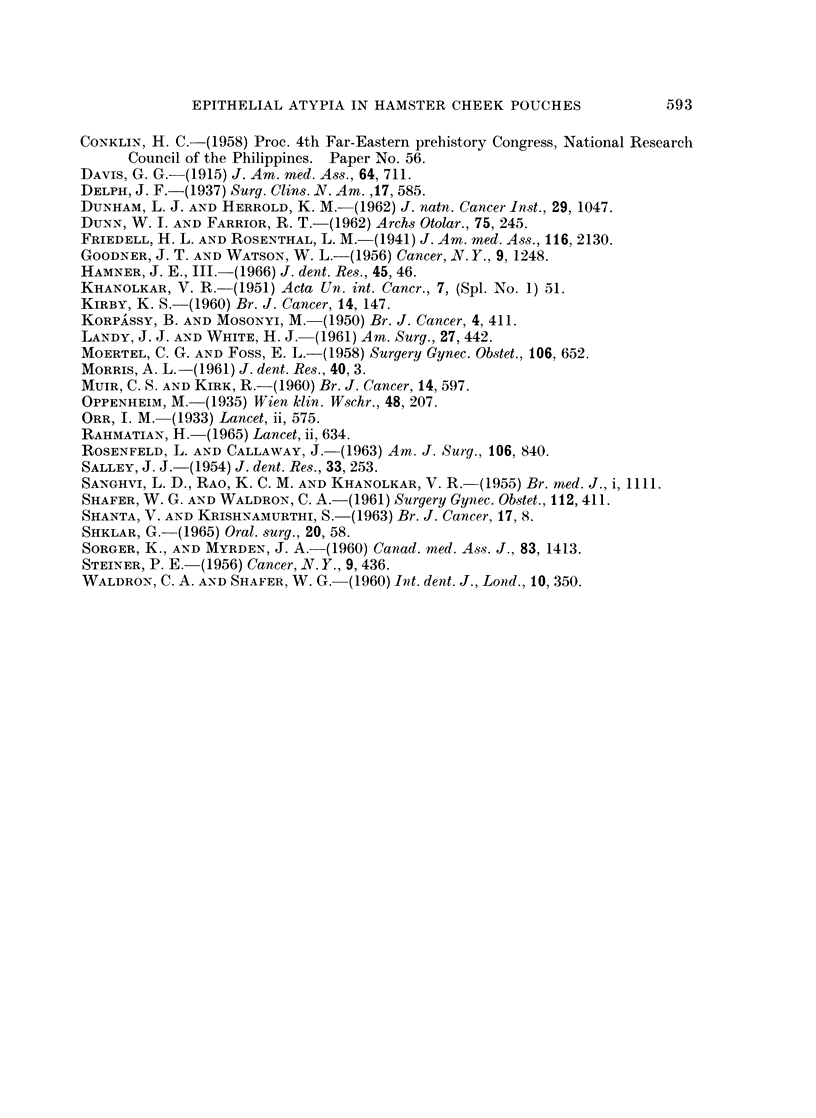# Epithelial atypia in hamster cheek pouches treated repeatedly with calcium hydroxide.

**DOI:** 10.1038/bjc.1966.73

**Published:** 1966-09

**Authors:** L. J. Dunham, C. S. Muir, J. E. Hamner

## Abstract

**Images:**


### EPITHELIAL ATYPIA IN HAMSTER CHEEK POUCHES TREATED REPEATEDLY WITH CALCIUM HYDROXIDE

LUCIA J. DUNHAM, C. S. MUIR AND J. E. HAMNER, III

*From the Laboratory of Pathology, National Cancer Institute, Bethesda, Maryland* 20014, *The Department of Pathology, University of Singapore, Singapore* 3, *and the National Institute of Dental Research, Bethesda, Maryland* 20014

Received for publication May 9, 1966

INVESTIGATORS have been interested for many years in the possibility that some oral cancers develop because of exposures to tobacco and betel quid chews (Friedell and Rosenthal, 1941 ; Orr, 1933). There is sound clinical and epidemiologic evidence that oral cancers are unusually frequent in persons who chew tobacco and " dip " snuff (Moertel and Foss, 1958 ; Sorger and Myrden, 1960 ; Landy and White, 1961 ; Dunn and Farrior, 1962 ; Rosenfeld and Callaway, 1963 ; Brown *et al.*, 1965). These cancers often develop at multiple sites in the mouth. Tobacco has been considered the ingredient of the betel quid most likely to cause changes in the oral mucosa that eventuate in the development of oral cancer (Khanolkar, 1951 ; Sanghvi, Rao and Khanolkar, 1955 ; Shanta and Krishnamurthi, 1963).

It has also been suggested that lime, a weak alkali which is almost always an ingredient of the betel quid, may act as a carcinogen or co-carcinogen (Davis, 1915 ; Orr, 1933 ; Atkinson *et al.*, 1964). Oral cancers are unusually frequent in New Guinea, and though lime is a component of the quid used there, tobacco is never added (Atkinson *et al.*, 1964). Conklin (1958) describes the Hanunóo Filipino native habit of betel chewing and comments in connection with undesirable effects of the chew : " Burning of the inner walls of the mouth and tongue because of an overdosage of lime, makes the mastication of salty foods and certain vegetables very painful." Another ingredient of the betel quid, gambier, which is an extract from the vine *Uncaria gambir*, is a suspected carcinogen by virtue of its tannin content (Korpássy and Mosonyi, 1950 ; Kirby, 1960). In experimental studies Muir and Kirk (1960) have induced cancers in the skin of two of twelve mice at the site of daily painting with an aqueous extract of a typical whole Singapore betel quid. However Dunham and Herrold (1962) could not induce tumors in the hamster cheek pouch treated by beeswax pellets that contained betel quid ingredients from several Asian countries.

In the present report the results are given of long-term exposure of the hamster cheek pouch to three ingredients of the betel quid administered in powdered form namely, calcium hydroxide, a proprietary brand of snuff, and gambier. Cornstarch dusting powder was used as a control.

### MATERIALS AND METHODS

Syrian hamsters (*Mesocricetus auratus*) were obtained at weaning from the Animal Production Center of the National Institutes of Health. The hamsters were fed Purina laboratory chow daily and small portions of carrot, apple, and kale three times a week. Drinking water was always available. Initially four to six animals were housed in wire cages but as they grew older they were housed alone to prevent cannibalism of cage-mates that died. There were both males and females in each experimental group.

The calcium hydroxide used was USP $Ca(OH)_2$ powder, Mallinckrodt, St. Louis, U.S.A. The brand " X " snuff was a " Scotch " or dry type prepared in the United States, and was one of two brands habitually used by a woman " snuff-dipper " who died as a result of multicentric oral cancer. The snuff was aromatic, but it is not known what the additives were, since snuffs are prepared by secret formulas. Cake gambier, obtained in Singapore, was ground to powder in a Waring Blendor. We used a cornstarch derivative dusting powder as a control material. This was a " Non-peptizable homogeneous mixture of amylose and amylopectin derived from cornstarch, together with 2 per cent magnesium oxide."

TABLE I.—*Survey of Experimental Groups of Hamsters, their Average Age, the Percentage Frequency of Cheek Pouch Lesions and the Incidental Tumours*

| Group* | Number in group | Average age in weeks | Percent with pouch lesions | Incidental tumours |
|---|---|---|---|---|
| I. Gambier alone | 14 | 73 | 14 | Lymphoma, bowel cancer, melanoma |
| II. Snuff alone | 7 | 99 | — | 2 with bowel cancer |
| III. Starch powder alone | 4 | 78 | — | —— |
| IV. $Ca(OH)_2$ alone† | 6 | 81 | 100 | 2 with bowel cancer |
| V. Equal parts $Ca(OH)_2$ and gambier† | 5 | 98 | 40 | 2 with lymphoma |
| VI. Equal parts $Ca(OH)_2$ and snuff | 6 | 77 | 100 | —— |
| VII. $Ca(OH)_2$ in a.m., snuff in p.m. | 6 | 67 | 100 | —— |
| VIII. $Ca(OH)_2$ in a.m., cornstarch in p.m. | 6 | 88 | 100 | Granulosa cell tumour of ovary |

* Animals in the first five groups received 250 mg. at each application for the first two weeks of the experiment.

† Treatments were reduced to 3 times each week between the second and 40th weeks of treatment.

There were eight groups of hamsters (Table I). Each of the four powdered substances (groups I to IV) and mixtures of equal parts of calcium hydroxide and gambier or snuff (groups V and VI) was administered for five consecutive days each week. Hamsters in groups VII and VIII were treated twice daily for five days each week. Hamsters in both these groups received calcium hydroxide in the morning, and three to five hours later in the afternoon, animals in group VII received snuff and animals in group VIII received cornstarch powder. A Vienna nasal speculum, child's size, was used to apply about 50 mg. of the test material at each treatment. The half-filled instrument was inserted deep in the right cheek pouch, the blades were opened, and the powder was distributed to the fundus and walls of the pouch and the inner surface of the lip. Thus both the pouch epithelium, which is similar to that lining the oral cavity but without accessory structures, and a part of the oral cavity were exposed to the powdered materials. A relatively prolonged contact is maintained in the cheek pouch. The powders were found plastered against its walls as long as six hours after application.

Treatment was started when the hamsters were three and a half to four and a half weeks old. They lived out their life spans and were either killed when moribund, or were found dead. Complete post mortem examinations were performed, tissues were fixed in buffered 10 per cent formalin, embedded in paraffin, and cut at 6 $\mu$. Sections were stained routinely with haematoxylin and eosin, and treated by the von Kossa method to demonstrate calcium.

### RESULTS

The pouches of 26 of the 29 hamsters treated with calcium hydroxide, either alone or in combination, showed gross changes (groups IV to VIII). The initial alkaline burn sometimes healed partially or completely between treatments, especially during the weekends when treatment was stopped, and in the early weeks of the experiment. Lesions were often multifocal and developed most often in the proximal third of the pouch, but were not restricted to this area. Chronically damaged pouches were inflamed and thickened and exhibited bleeding points, shallow ulcers, abscesses, or scars that resulted in puckering of the pouch wall and sometimes led to sloughing of tissues and a shortened pouch. In some instances portions of the pouch wall appeared to be nodular, and in others thin crusts covered some of the lesions. Occasionally the mucosal surface of the lip was grossly inflamed. A fundal scar was noted in the pouch of one hamster treated with gambier. There were no changes in the pouches of hamsters treated with snuff alone or with starch powder alone (Table I).

Microscopic examination of tissue from pouches of the 26 affected hamsters treated with calcium hydroxide revealed one or more of the following lesions in the lamina propria : deposits of calcium, infiltration of inflammatory cells, giant cells, and fibroblastic proliferation (Fig. 1 to 4). The various lesions noted in the

---

### EXPLANATION OF PLATES

FIG. 1.—Cheek pouch of hamster treated with calcium hydroxide for 81 weeks. Lesions were multifocal. The epithelium at the edge of a small ulcer is atrophic. Chronic inflammatory cells are scattered throughout the lamina propria, where a single darkstaining deposit of calcium is seen. H. and E. $\times 205$.

FIG. 2.—Cheek pouch of hamster treated with equal parts of calcium hydroxide and brand " X " snuff for 74 weeks. The epithelium is atrophic. There is no cellular reaction about the clumped deposits of calcium situated deep in the lamina propria. H. and E. $\times 80$.

FIG. 3.—Biopsy of a multifocal inflamed nodular lesion that developed in the cheek pouch of a hamster treated with calcium hydroxide for 42 weeks (life, 61 weeks). The epithelium shows hyperplasia, hyperkeratosis and acanthosis. The underlying connective tissue exhibits fibrosis, a deposit of calcium and giant cells. H. and E. $\times 170$.

FIG. 4.—Cheek pouch of hamster treated with calcium hydroxide in the morning and brand " X " snuff in the afternoon for 81 weeks. Lesions were multifocal. Hyperkeratosis, acanthosis, rete peg formation and basal cell hyperplasia are seen in the epithelium, and there is a multinucleated giant cell in the lamina propria (arrow). H. and E. $\times 200$.

FIG. 5.—Cheek pouch of hamster treated with calcium hydroxide in the morning and cornstarch powder in the afternoon, for 102 weeks. The thickened pouch wall shows epithelial hyperplasia, hyperkeratosis, acanthosis and rete peg formation. H. and E. $\times 135$.

FIG. 6.—Another area of the cheek pouch shown in Fig. 1, treated with calcium hydroxide. This is a focus of epithelial hyperplasia, parakeratosis and cellular atypia. Note the loss of polarity, hyperchromatism and disturbed basal cell layer. H. and E. $\times 205$.

FIG. 7.—Cheek pouch of hamster treated with equal parts of calcium hydroxide and gambier for 121 weeks. Lesions were multifocal. Atypical cells show loss of polarity, and some basal cells are fusiform. H. and E. $\times 205$.

Dunham, Muir and Hamner.

Dunham, Muir and Hamner.

epithelium were ulceration, foci of inflammatory cells, atrophy or hyperplasia, hyperkeratosis, parakeratosis, acanthosis, and cellular atypia (Fig. 1 to 7). The lesion illustrated in Fig. 2 probably followed a small ulcer that permitted the entry of calcium to the lamina propria ; after healing the calcific deposit was covered by atrophic epithelium. Hyperplasia, shown by increased thickness of the epithelium, and rete pegs (Fig. 4 and 5) are not normally seen in the hamster pouch. The lesions most suggestive of a pre-neoplastic condition were small foci of atypical cells (Fig. 6 and 7) which showed loss of cellular polarity, and cells in the basal layer that were hyperchromatic and fusiform.

The tongue of a hamster that received calcium hydroxide in the morning and snuff in the afternoon showed the usual features of a chemical burn, i.e. a large ulcer, epithelial atrophy, homogenized collagen in the lamina propria, chronic inflammatory infiltrate, and necrosis of some of the muscle bundles. Enlarged submaxillary lymph nodes in four hamsters were hyperplastic on microscopic examination. Three of the hamsters had been treated with lime, and one with cornstarch powder. The forestomachs of the animals that had received calcium hydroxide alone or in combination were usually normal, though two showed slight hyperkeratosis and epithelial hyperplasia. In comparison with the calcium hydroxide, the other substances caused little damage. Microscopic lesions at single foci in the pouches of 2 of the 14 hamsters treated with gambier were focal infiltration of inflammatory cells in one instance and a minute ulcer in the other. Focal inflammation was seen also in the outer lips of two hamsters treated with gambier. A polyp of the forestomach that developed in a hamster treated with gambier alone was probably spontaneous, and the glandular stomachs and other organs of the hamsters in the experiment were within normal limits. The types and distribution of the incidental tumours that developed in hamsters in five of the experimental groups (Table I) resemble the spontaneous tumors of hamsters described by Dunham and Herrold (1962).

### DISCUSSION

In the present study calcium hydroxide apparently entered the cheek pouch wall through a break in its surface and calcium accumulated deep in the connective tissue. There was little cellular reaction to the clumped deposits of calcium. Oppenheim (1935) treated the skin of a rabbit with calcium chloride and has published a microphotograph showing masses of calcium in the lamina propria and epithelial acanthosis. It is of interest that one of the authors (J.E.H.) observed calcium deposits in the connective tissue beneath a squamous cell carcinoma of the buccal mucosa in a Vietnamese female betel quid chewer (Armed Forces Institute of Pathology, accession No. 1181731). Betel quids usually contain calcium hydroxide. The deposits below the cancerous epithelium resembled those seen in hamster pouches treated with lime, though they were comparatively small and scattered.

At least three of the lesions in the pouch epithelium produced by repeated applications of calcium hydroxide progressed to distinct cellular atypia. The lesions resembled oral dyskeratosis (leukoplakia) in man (Waldron and Shafer, 1960 ; Shafer and Waldron, 1961 ; Shklar, 1965), except that they were focal, and did not widely involve the cheek pouch in any instance. We did not consider that these lesions were pre-invasive cancer.

Carcinogenesis in the cheek pouch of hamsters by painting with 7,12-dimethylbenz(*a*)anthracene (DMBA) proceeds through inflammatory, degenerative, regenerative and hyperplastic phases (Salley, 1954 ; Morris, 1961 ; Hamner, 1966). The similar lesions produced by calcium hydroxide progressed only to the beginning of the hyperplastic phase. The treated hamsters lived to the normal or nearly normal extent of their life spans. It cannot be ascertained from this experiment whether the lesions that developed were the final phase of the reaction to the treatment with calcium hydroxide, or whether they had the potential of progression to neoplasia. To assist in answering these questions calcium hydroxide is being applied to pouches using a wetting agent as vehicle, and pouches of hamsters maintained on a modified diet are being treated with calcium hydroxide.

Oral cancers are relatively frequent in betel quid chewers. The relationship of these cancers to the lime or calcium hydroxide that is added to the quids is uncertain. Perhaps damage such as is produced in the hamster pouch by repeated applications of calcium hydroxide renders the affected area more susceptible to the effects of a carcinogen. It is known that oesophageal cancer sometimes develops long after oesophageal stricture resulting from burns due to the ingestion of sodium hydroxide (lye) (Delph, 1937 ; Bigger and Vinson, 1950). Reports from Iran suggest that cancer of the oesophagus is related to the habit of chewing *nass* (Azarmie, 1965, personal communication ; Rahmatian, 1965). The ingredients of *nass* are lime, tobacco, and wood chips or wood ash. It has been postulated that similar environmental factors may predispose to cancers of the mouth, oesophagus, and tissues of the upper gastrointestinal tract in general (Goodner and Watson, 1956 ; Steiner, 1956).

The observation in the present experiment that powdered tobacco (snuff) did not produce lesions in the hamster cheek pouch does not prove that tobaccos cannot cause injury of the human buccal mucosa that sometimes results in oral cancer. There is no sound experimental proof that tobaccos induce cancer in the mouth, yet it is well recognized that this cancer develops with unusual frequency in " snuff-dippers " and in persons who chew tobacco.

### SUMMARY

Repeated applications of calcium hydroxide (lime) severely injured the hamster cheek pouch. Three of the inflammatory and hyperplastic lesions that developed in the pouches of 26 treated hamsters progressed to epithelial atypia. Powdered tobacco (snuff) did not alone produce lesions, and a dusting powder (cornstarch derivative) did not produce lesions. Inflammatory lesions that developed in 2 of 14 pouches treated with gambier were minimal. The effects of calcium hydroxide were not enhanced when snuff was applied in a mixture with the calcium hydroxide or when snuff or cornstarch powder was applied several hours after treatment with calcium hydroxide.

### REFERENCES

Atkinson, L., Chester, I. C., Smyth, F. G. and Ten Seldam, R. E. J.—(1964) *Cancer, N.Y.*, **17**, 1289.

Bigger, I. A. and Vinson, P. P.—(1950) *Surgery, St. Louis*, **28**, 887.

Brown, R. L., Suh, J. M., Scarborough, J. E., Wilkins, S. A., Jr. and Smith, R. R.—(1965) *Cancer, N.Y.*, **18**, 2.

CONKLIN, H. C.—(1958) Proc. 4th Far-Eastern prehistory Congress, National Research Council of the Philippines. Paper No. 56.
DAVIS, G. G.—(1915) *J. Am. med. Ass.*, **64**, 711.
DELPH, J. F.—(1937) *Surg. Clins. N. Am.* ,**17**, 585.
DUNHAM, L. J. AND HERROLD, K. M.—(1962) *J. natn. Cancer Inst.*, **29**, 1047.
DUNN, W. I. AND FARRIOR, R. T.—(1962) *Archs Otolar.*, **75**, 245.
FRIEDELL, H. L. AND ROSENTHAL, L. M.—(1941) *J. Am. med. Ass.*, **116**, 2130.
GOODNER, J. T. AND WATSON, W. L.—(1956) *Cancer, N.Y.*, **9**, 1248.
HAMNER, J. E., III.—(1966) *J. dent. Res.*, **45**, 46.
KHANOLKAR, V. R.—(1951) *Acta Un. int. Cancr.*, **7**, (Spl. No. 1) 51.
KIRBY, K. S.—(1960) *Br. J. Cancer*, **14**, 147.
KORPÁSSY, B. AND MOSONYI, M.—(1950) *Br. J. Cancer*, **4**, 411.
LANDY, J. J. AND WHITE, H. J.—(1961) *Am. Surg.*, **27**, 442.
MOERTEL, C. G. AND FOSS, E. L.—(1958) *Surgery Gynec. Obstet.*, **106**, 652.
MORRIS, A. L.—(1961) *J. dent. Res.*, **40**, 3.
MUIR, C. S. AND KIRK, R.—(1960) *Br. J. Cancer*, **14**, 597.
OPPENHEIM, M.—(1935) *Wien klin. Wschr.*, **48**, 207.
ORR, I. M.—(1933) *Lancet*, ii, 575.
RAHMATIAN, H.—(1965) *Lancet*, ii, 634.
ROSENFELD, L. AND CALLAWAY, J.—(1963) *Am. J. Surg.*, **106**, 840.
SALLEY, J. J.—(1954) *J. dent. Res.*, **33**, 253.
SANGHVI, L. D., RAO, K. C. M. AND KHANOLKAR, V. R.—(1955) *Br. med. J.*, i, 1111.
SHAFER, W. G. AND WALDRON, C. A.—(1961) *Surgery Gynec. Obstet.*, **112**, 411.
SHANTA, V. AND KRISHNAMURTHI, S.—(1963) *Br. J. Cancer*, **17**, 8.
SHKLAR, G.—(1965) *Oral. surg.*, **20**, 58.
SORGER, K., AND MYRDEN, J. A.—(1960) *Canad. med. Ass. J.*, **83**, 1413.
STEINER, P. E.—(1956) *Cancer, N.Y.*, **9**, 436.
WALDRON, C. A. AND SHAFER, W. G.—(1960) *Int. dent. J., Lond.*, **10**, 350.